# Obesity Paradox of All-Cause Mortality in 4,133 Patients Treated with Coronary Revascularization

**DOI:** 10.1155/2021/3867735

**Published:** 2021-11-18

**Authors:** Chengzhuo Li, Didi Han, Fengshuo Xu, Shuai Zheng, Luming Zhang, Zichen Wang, Rui Yang, Haiyan Yin, Jun Lyu

**Affiliations:** ^1^Intensive Care Unit, The First Affiliated Hospital of Jinan University, Guangzhou 510630, Guangdong Province, China; ^2^School of Public Health, Xi'an Jiaotong University Health Science Center, Xian 710061, Shaanxi, China; ^3^School of Public Health, Shaanxi University of Chinese Medicine, Xianyang 712046, Shaanxi, China; ^4^Department of Public Health, University of California, Irvine 92697, California, USA

## Abstract

**Objectives:**

The purpose of this study was to determine whether there is a dose-response relationship between body mass index (BMI) and all-cause mortality in patients after coronary revascularization.

**Methods:**

The MIMIC-III database (version 1.4) was used as the sample population. For variables with less than 10% of values missing, we used the mice package of R software for multiple imputations. Cox regression was used to determine the risk factors of all-cause mortality in patients. RCSs were used to observe the relationship between BMI and all-cause mortality. Additional subgroup and sensitivity analyses were also performed to explore whether the conclusion can be applied to specific groups.

**Results:**

Both univariate and multivariate Cox models indicated that the mortality risk was lower for overweight patients than for normal-weight patients (*P* < 0.05). In RCS models, BMI had a U-shaped relationship with all-cause mortality of patients after coronary artery bypass grafting (CABG) (*P* for nonlinearity = 0.0028). There was a weak U-shaped relationship between BMI and all-cause mortality after percutaneous coronary intervention (PCI), but the nonlinear relationship between these two parameters was not significant (*P* for nonlinearity = 0.1756).

**Conclusions:**

The obesity paradox does exist in patients treated with CABG and PCI. RCS analysis indicated that there was a *U*-shaped relationship between BMI and all-cause mortality in patients after CABG. After sex stratification, the relationship between BMI and all-cause mortality in male patients who received PCI was *L*-shaped, while the nonlinear relationship among females was not significant.

## 1. Introduction

Coronary artery revascularization is an important method for treating coronary heart disease. Coronary artery bypass grafting (CABG) and percutaneous coronary intervention (PCI) are the two most common operations in coronary revascularization. The body mass index (BMI) is a prognostic factor for all-cause mortality in many diseases, with the prognosis being better in people with a normal BMI than in those who are overweight or obese [1, 2]. However, studies have indicated that some overweight or even obese patients have a better prognosis than those with a normal BMI, which is referred to as the obesity paradox [3, 4].

Gruberg et al. were the first to mention the obesity paradox in patients after revascularization [5]. That study found that patients with a normal BMI were found to have higher hospitalization and 1-year mortality rates than those who were overweight or obese. This indicates that being overweight or obese may protect patients and reduce mortality. However, some studies have attributed the obesity paradox to factors other than BMI [6, 7].

The relationship between BMI and all-cause mortality in patients after coronary revascularization remains unclear. Most studies have included BMI as a continuous or categorical variable when performing logistic or Cox regression analysis, which does illustrate changes in BMI effectively [8–10]. However, basic research is still needed to explore this relationship.

The purpose of this study was to determine whether there is a dose-response relationship between BMI and all-cause mortality in coronary heart disease patients undergoing coronary artery revascularization, thereby determining whether there is an obesity paradox in the prognosis of patients who underwent coronary artery revascularization. We hypothesized that it is a nonlinear relationship and used restricted cubic splines (RCSs) to determine the dose-response relationship.

## 2. Methods

### 2.1. Study Design and Population

The MIMIC-III database contains data on 53,423 adult patients (16 years or older) admitted to an intensive care unit between 2001 and 2012. The MIMIC-III database (version 1.4) was sampled, and the project was approved by the Institutional Review Boards of the Beth Israel Deaconess Medical Center (Boston, Massachusetts) and the Massachusetts Institute of Technology (Cambridge, Massachusetts). Consent was not required from individual patients since all of the protected health information of the project has been deidentified [11, 12]. We completed recognized courses for protecting human research participants, including the requirements of the HIPAA (Health Insurance Portability and Accountability Act), and signed a data usage agreement. The Institutional Review Boards of the Beth Israel Deaconess Medical Center and Massachusetts Institute of Technology have approved the use of the MIMIC-III database by any researcher who meets the data user requirements, and the requirement for patient's informed consent is waived.

We used the ninth edition of the International Classification of Diseases (Clinical Modification) codes to identify and analyze all patients in the MIMIC-III database whose primary operation was revascularization (including CABG and PCI). BMI was calculated using the heights and weights recorded in the database. As the main study indicator, all missing values for BMI were deleted. The MIMIC-III database is connected to the social security database to record the follow-up times and outcomes of patients. Our study outcome was all-cause mortality as registered by the social security bureau of the patient. The follow-up times were reported in days.

**Figure 1:**
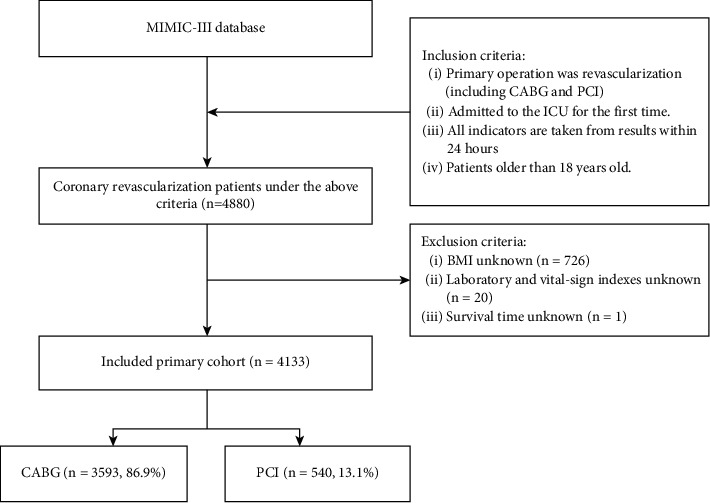
The screening flowchart.

We also extracted patient demographic, laboratory, and vital-sign indicators from the database. These factors were possible confounding factors in the relationship between BMI and all-cause mortality. All indicators were extracted from the diagnoses_icd, admissions, patients, icustays, labevents, and procedures_icd parameters in the database. The flowchart for data inclusion and exclusion is displayed in Figure 1.

The purpose of our research was to determine the dose-response relationship between BMI and all-cause mortality in patients after revascularization and to determine the existence of the obesity paradox while adjusting for possible confounding factors.

### 2.2. Statistical Analysis

All data were extracted using the Structured Query Language (SQL). Since BMI was the main study indicator, all missing values were deleted, and other possible confounding factors with >10% of values missing were also deleted. We used the mice package of R software for multiple imputation for variables with <10% of values missing [13]. This package uses relevant random samples based on the distribution of predicted values to replace the missing values with estimated values. We constructed a dataset by merging five imputation datasets.

The World Health Organization (WHO) standards were used to divide BMI into underweight, normal weight, overweight, and obese categories. Age was divided into three categories: youth, middle age, and elderly. All categorical variables were expressed as numbers and percentages, and the chi-squared and Fisher's exact tests were used to determine the differences between the two groups. All continuous variables were expressed as medians and interquartile-range values, and the Mann–Whitney *U* test was used to identify differences between the two groups.

The Kaplan–Meier curve and log-rank test were used to evaluate whether there are differences in survival rates between different operations. Cox regression was used to determine the risk factors of patient all-cause mortality. We constructed univariate models, a demographic adjustment model (model I), and a multivariate adjustment model (model II). A trend test was used to determine linear trends between BMI and all-cause mortality.

RCSs were used to assess the nonlinear relationship between BMI and all-cause mortality. We divided the distribution of BMI into quartiles as the knots of the nonlinear models. We analyzed the nonlinear relationship between BMI and patient all-cause mortality and determined the *P* value of the nonlinear test.

We also performed a subgroup analysis of sex and age to determine whether a relationship existed within a specific subgroup. We performed the following sensitivity analyses to determine the stability of our results: (1) we removed all patients who died within 30 days of their operation and performed the RCS analysis again, (2) abnormal BMI values (more than three times the standard deviation) were deleted before the analysis, and (3) we deleted the missing values from the original data before performing multiple imputations and reanalyzed the data to explore the difference resulting from multiple imputations.

All statistical analyses were conducted using Navicat Premium and R software (version 3.6.2, The R Foundation for Statistical Computing, Vienna, Austria). All cited *P* values were two-sided, and *P* < 0.05 was considered statistically significant.

### 2.3. Patient and Public Involvement

This research was done without patient involvement. Patients were not invited to comment on the study design and were not consulted to develop patient-relevant outcomes or interpret the results. Patients were not invited to contribute to the writing or editing of this document for readability or accuracy.

## 3. Results

### 3.1. Population Characteristics

**Figure 2:**
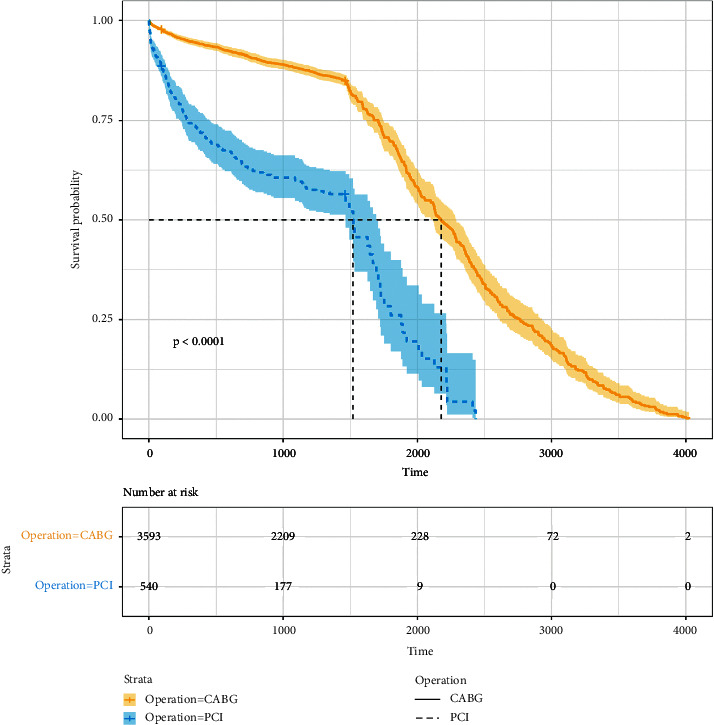
Kaplan–Meier survival curves for all-cause mortality.

Our final sample comprised 4133 patients treated with coronary artery revascularization in the MIMIC-III database: CABG patients (86.9%, *n* = 3593) and PCI patients (13.1%, *n* = 540). Their baseline data are listed in Table 1, which indicates that most of the variables differed significantly between the two types of surgery (*P* < 0.05). Table 1 indicates that there was a very small number of underweight patients, with overweight patients comprising the largest proportion (approximately 39%). Most patients were male, married, and white.  Figure 2 displays the survival curves after surgery for both types of patients. The figure suggests that there are significant statistical differences between the two, and so the two types of patients were analyzed separately.

### 3.2. Univariate and Multivariate Cox Regression Analyses

We first divided BMI into four categories based on the WHO standards and then performed univariate and multivariate Cox regression analyses with normal weight as the reference. After adjusting all of the variables, the Cox regression model showed that length of stay, Elixhauser Comorbidity Index (ECI), urine output, pH, potassium, prothrombin time, red blood cell distribution width (RDW), age, marital status, and BMI were the significant prognostic factors for CAGB patients, while ventilator support, ECI, urine output, heart rate, temperature, hematocrit, RDW, sex, and BMI were the significant prognostic factors for PCI patients. The Cox regression results are presented in detail in Supplementary Tables S1 and S2.

Tables 2 and 3 present the results for model I (with the demographic characteristics of age, sex, race, and marital status adjusted) and model II (with the risk factors derived from the multivariate Cox analysis adjusted).  Table 2 lists the hazard ratio (HR) and 95% confidence interval (CI) values for CABG patients according to their BMI group. Both the univariate model and model II showed that the risk of mortality was lower for overweight patients than for normal-weight patients (*P* < 0.05).  Table 3 lists the HRs and 95% CIs for PCI patients. All three models indicated that the mortality risk was lower for overweight PCI patients than for normal-weight patients (*P* < 0.05). The trend tests of the six models revealed that a significant linear trend was only present for the BMI univariate model of PCI patients (*P*=0.002), which also suggested the presence of a nonlinear relationship between BMI and all-cause mortality.

### 3.3. RCS Analyses of Nonlinear Relationships

Supplementary Tables S3 and S4 list the HR and 95% CIs of the BMI quartiles for all-cause mortality. The first quartile (Q1) was taken as a reference group for comparison with the other groups to obtain the corresponding HRs. In CABG patients, compared with the Q1 of BMI, the HR for Q2 of BMI was 0.722 (95% CI = 0.586–0.888) for the univariate model, 0.759 (95% CI = 0.616–0.936) for model I, and 0.740 (95% CI = 0.599–0.913) for model II (all *P* < 0.05). Unlike CABG patients, the Q2 group of PCI patients did not show significant differences, whereas the Q3 group did show significant differences. Compared with the Q1 of BMI, the HR for Q3 of BMI was 0.506 (95% CI = 0.332–0.773) for the univariate model, 0.572 (95% CI = 0.370–0.884) for model I, and 0.601 (95% CI = 0.392–0.924) for model II (all *P* < 0.05).

**Figure 3:**
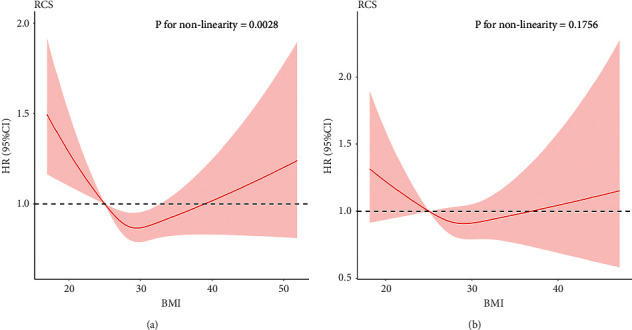
Dose-response curves for BMI and all-cause mortality of CABG patients (a) and PCI patients (b).

We constructed RCS models to further analyze the relationship between BMI and patient mortality.  Figure 3 shows the dose-response curves of BMI and all-cause mortality after adjusting for the factors that were significant in the Cox analysis. The dose-response analysis revealed a U-shaped curve between BMI and the risk of all-cause mortality in CABG patients (*P* for nonlinearity = 0.0028) (Figure 3(a)). In contrast, there was a weak U-shaped relationship between BMI and all-cause mortality after PCI, but no significant nonlinear relationship between these two variables (*P* for nonlinearity = 0.1756) (Figure 3(b)).

### 3.4. Subgroup Analyses

After adjusting for the corresponding confounding factors, the subgroup analysis of BMI and all-cause mortality of patients after CABG (Supplementary Table S5) showed that there was no significant interaction effect of sex (*P* for interaction = 0.330) or age (*P* for interaction = 0.883). This indicates that the relationship between BMI and CABG did not differ significantly with sex or age. However, Supplementary Table S6 showed that significant interaction was found in the PCI group for stratification according to sex (*P* for interaction = 0.006). This suggests that the impact of BMI on the mortality of PCI patients is affected by sex.

**Figure 4:**
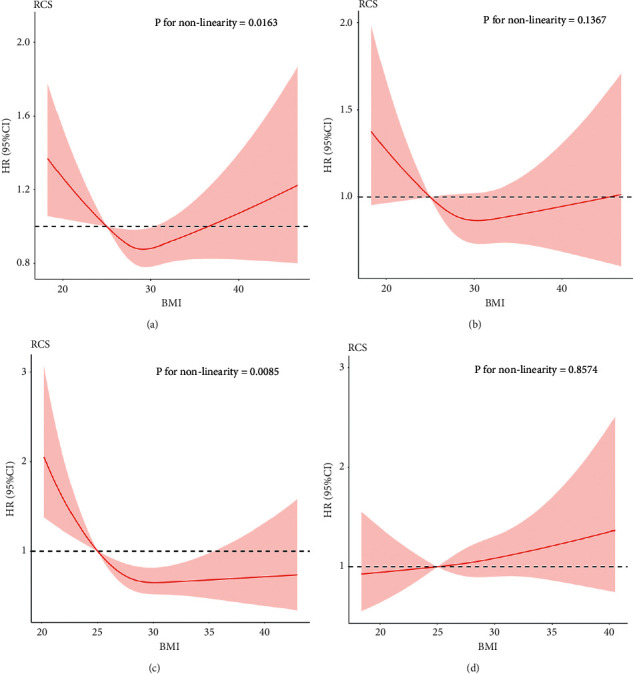
Dose-response curves for BMI and all-cause mortality of CABG patients by sex group with male (a) and female (b); dose-response curves for BMI and all-cause mortality of PCI patients by sex group with male (c) and female (d).

We, therefore, constructed RCS models to perform a sex-stratified analysis. As shown in Figure 4, the dose-response relationship appeared as *U*-shaped curves for all-cause mortality of BMI and CABG. However, the nonlinear trend was significant for males (*P* for nonlinearity = 0.0163) but not for females (*P* for nonlinearity = 0.1367). The sex-related difference appeared to be greater in PCI patients. There was an approximate L-shaped relationship between BMI and all-cause mortality in male patients (*P* for nonlinearity = 0.0085) but not in female patients (*P* for nonlinearity = 0.8574).

### 3.5. Sensitivity Analysis

**Figure 5:**
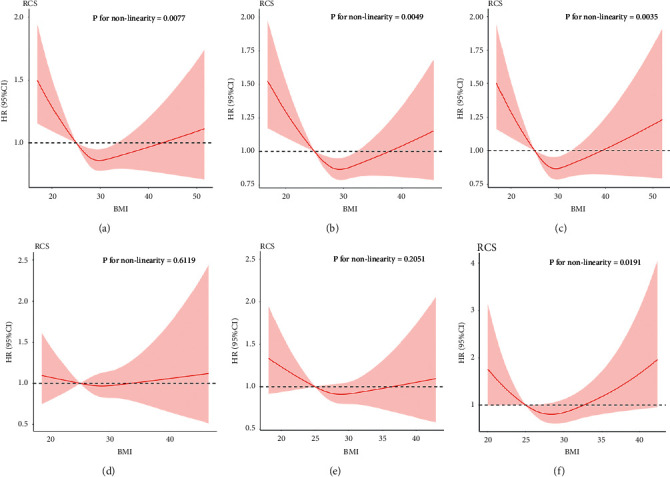
Sensitivity analysis of (1) excluding patients who died within 30 days after the CABG (a) and PCI (d); (2) excluding patients who had abnormal BMI values of CABG patients (b) and PCI patients (e); (3) the complete data before performing multiple imputation of CABG patients (c) and PCI patients (f).

After excluding patients who died within 30 days after the operation or had abnormal BMI values, the RCS analysis showed that the nonlinear relationship between BMI and all-cause mortality remained consistent with the previous results. That is, there was a significant *U*-shaped nonlinear relationship in CABG patients, but no significant nonlinear relationship in PCI patients. However, analyzing the complete data before performing multiple imputations revealed a *U*-shaped relationship between BMI and all-cause mortality for both CABG and PCI patients (*P* for nonlinearity <0.05) (Figure 5). It should be noted that there was no significant nonlinear relationship for PCI patients after performing multiple imputation.

## 4. Discussion

Higher BMI is usually a risk factor for adverse outcomes of cardiovascular disease and various complications [14]. However, many studies have found that overweight or obese people have a survival advantage compared to people who are overly thin or have a BMI within the normal range, which is called the obesity paradox [15, 16]. There have been some reports on the obesity paradox in patients after revascularization. However, most of these studies simply divided BMI into four categories according to international standards and segmented the relationship between BMI and outcome [17, 18]. In contrast, the present study used RCSs to explore the nonlinear relationship between BMI and all-cause mortality after coronary revascularization surgery and, thereby, expressed the data using continuous and smooth graphs, which is more intuitive for explaining the overall trend for ORs of BMI than using the traditional segmented analysis.

The present results show that the survival rate is higher for CABG patients than for PCI patients. In univariate and multivariate Cox regression analyses, BMI was a prognostic factor for all-cause mortality in patients regardless of whether they received CABG or PCI. In the RCS analysis, the models before and after adjustment all showed a *U*-shaped relationship between the BMI and all-cause mortality for CABG patients. For PCI patients, there was a weak *U*-shaped relationship between these variables, but no significant nonlinear relationship between them after performing multivariate adjustment.

It is especially noteworthy that our subgroup analysis of PCI patients revealed a significant interaction between sex and BMI, which means that sex may affect the relationship between BMI and mortality. There was no significant difference in the Cox regression results for the relationship between BMI and all-cause mortality among females. Correspondingly, in the RCS analysis, we only observed significant nonlinear relationships in males, which indicates that the *U*-shaped relationship may only apply to males and that further investigations are needed into the relationship for females.

Previous studies have found BMI to be a poor discriminator of females with different risks of coronary heart disease. Weight and BMI are now considered less important than previously thought [19]. Materko et al. showed that the existing BMI classification standards are not suitable for females, [20] which might explain why the relationship between BMI and all-cause mortality in females was not significant in the present study.

In addition, after removing all missing data without performing multiple imputations in the sensitivity analysis, the nonlinear relationship became significant in PCI patients. This result might have been due to there being too many missing values for PCI patients since only 97 patients remained after removing the missing values. Compared with the 3345 CABG patients, the much smaller sample for PCI patients might have introduced bias into the study. Therefore, the relationship between BMI and all-cause mortality in PCI patients needs to be investigated further with large samples in prospective cohorts. The results of the other sensitivity analysis are consistent with the results obtained when analyzing the full sample, which further confirms the present research conclusions.

The present research has yielded further evidence for the existence of the obesity paradox. However, unlike previous studies [21–23], we used RCSs to obtain HR values, and hence, more-precise risk ranges. In CABG patients, taking a BMI of 25 kg/m^2^ as the reference value, patients with a BMI within the range of 25–32 kg/m^2^ had a lower HR, which corresponds to a higher survival rate. The reasons for this obesity paradox remain to be elucidated, but some possible explanations are given below. One hypothesis is that obese patients receive more aggressive drug treatment than normal-weight patients, resulting in better control of their various indicators [24, 25]. In addition, it has been reported that compared with normal-weight patients, obese hypertensives have a higher cardiac output, enlarged blood volume, and lower systemic vascular resistance. This means that, for a given level of arterial pressure, obese patients will have lower total peripheral resistance than lean patients, which could improve the survival rate of obese patients [26, 27]. It has also been shown that BMI is a poor indicator for categorizing obesity. Indicators such as waist circumference and waist-to-height ratio may better reflect the true degree of obesity. [28, 29].

This study was subject to some limitations. Firstly, the MIMIC-III database is from a single center, and so the present research results need to be verified in a multicenter population. Secondly, although we used Cox regression for analysis, cross-sectional studies cannot be used to determine causality, and so future prospective cohort studies are needed. Thirdly, the large number of missing values for PCI patients may have made the results unstable. Larger samples are needed to confirm the conclusions and sex differences for PCI patients.

## 5. Conclusion

The analysis performed in this study of the MIMIC-III database revealed that the obesity paradox does exist in patients treated with CABG and PCI. The RCS analysis showed that BMI has a *U*-shaped relationship with all-cause mortality in CABG patients, while this relationship is not significant in PCI patients. However, sex stratification revealed an *L*-shaped relationship between BMI and all-cause mortality in male PCI patients, while there was no significant nonlinear relationship in females. Multicenter and large-sample prospective studies are needed to further explore sex differences in the obesity paradox.

## Figures and Tables

**Table 1:** Baseline characteristics.

Variables	Total (*n* = 4133)	CABG (*n* = 3593)	PCI (*n* = 540)	*P* value
Age (*n*) (%)				<0.001
Youth	1847 (44.69)	1601 (44.56)	246 (45.56)	
Middle aged	1733 (41.93)	1567 (43.61)	166 (30.74)	
The elder	553 (13.38)	425 (11.83)	128 (23.70)	
Sex (*n*) (%)				<0.001
Male	3113 (75.32)	2751 (76.57)	362 (67.04)	
Female	1020 (24.68)	842 (23.43)	178 (32.96)	
Race (*n*) (%)				<0.001
White	2840 (68.72)	2446 (68.08)	394 (72.96)	
Black	131 (3.17)	100 (2.78)	31 (5.74)	
Asian	83 (2.01)	76 (2.12)	7 (1.30)	
Hispanic or Latino	101 (2.44)	92 (2.56)	9 (1.67)	
Others	978 (23.66)	879 (24.46)	99 (18.33)	
Insurance (*n*) (%)				0.014
Government	83 (2.01)	70 (1.95)	13 (2.41)	
Medicaid	183 (4.43)	163 (4.54)	20 (3.70)	
Medicare	2241 (54.22)	1915 (53.30)	326 (60.37)	
Private	1610 (38.95)	1432 (39.86)	178 (32.96)	
Self-pay	16 (0.39)	13 (0.36)	3 (0.56)	
Marital (*n*) (%)				<0.001
Married	2674 (64.70)	2368 (65.91)	306 (56.67)	
Unmarried	559 (13.53)	461 (12.83)	98 (18.15)	
DSW	729 (17.64)	609 (16.95)	120 (22.22)	
Others	171 (4.14)	155 (4.31)	16 (2.96)	
BMI (*n*) (%)				0.001
I: normal	953 (23.06)	817 (22.74)	136 (25.19)	
II: underweight	46 (1.11)	32 (0.89)	14 (2.59)	
III: overweight	1618 (39.15)	1406 (39.13)	212 (39.26)	
IV: obesity	1516 (36.68)	1338 (37.24)	178 (32.96)	
Length of stay (day), median (IQR)	2.07 (1.20, 3.30)	2.10 (1.20, 3.29)	1.90 (1.15, 3.67)	0.165
ECI, median (IQR)	6.00 (−2.00, 13.00)	6.00 (−2.00, 11.00)	9.00 (1.00, 19.00)	<0.001
Urine output (ml), median (IQR)	2088.00 (1510.00, 2858.00)	2110.00 (1543.00, 2858.00)	1912.50 (1268.75, 2833.75)	<0.001
Heartrate (min-1), median (IQR)	84.00 (77.73, 90.48)	84.77 (78.97, 90.91)	75.31 (67.71, 85.28)	<0.001
SBP (mmHg), median (IQR)	112.08 (106.53, 119.53)	111.77 (106.58, 118.81)	115.06 (106.03, 125.23)	<0.001
DBP (mmHg), median (IQR)	57.15 (53.08, 61.83)	56.71 (52.83, 60.93)	62.48 (55.84, 69.78)	<0.001
Mean BP (mmHg), median (IQR)	74.82 (71.07, 79.33)	74.59 (71.07, 78.69)	77.56 (71.01, 83.59)	<0.001
Resprate (min-1), median (IQR)	16.83 (15.30, 18.78)	16.68 (15.17, 18.54)	18.17 (16.31, 20.25)	<0.001
Temperature (°C), median (IQR)	36.82 (36.52, 37.14)	36.83 (36.53, 37.15)	36.69 (36.43, 37.03)	<0.001
SpO_2_ (%), median (IQR)	98.13 (97.13, 98.96)	98.23 (97.30, 99.00)	97.14 (95.97, 98.29)	<0.001
Free calcium (mmol/L), median (IQR)	1.15 (1.12, 1.19)	1.15 (1.12, 1.19)	1.13 (1.09, 1.18)	<0.001
pCO_2_ (mmHg), median (IQR)	41.00 (37.00, 45.00)	41.00 (38.00, 45.00)	41.50 (37.00, 46.00)	0.232
pH, median (IQR)	7.40 (7.37, 7.44)	7.41 (7.38, 7.44)	7.38 (7.33, 7.42)	<0.001
pO_2_ (mmHg), median (IQR)	380.00 (269.00, 433.00)	390.00 (311.00, 437.00)	155.00 (88.00, 338.25)	<0.001
Anion gap (mEq/L), median (IQR)	13.00 (11.00, 15.00)	13.00 (11.00, 15.00)	15.00 (13.00, 17.25)	<0.001
Bicarbonate (mEq/L), median (IQR)	25.00 (23.00, 27.00)	25.00 (23.00, 27.00)	24.00 (21.75, 26.00)	<0.001
Chloride (mEq/L), median (IQR)	105.00 (102.00, 108.00)	105.00 (102.00, 109.00)	103.00 (100.00, 106.00)	<0.001
Creatinine (mEq/L), median (IQR)	0.90 (0.80, 1.20)	0.90 (0.80, 1.10)	1.10 (0.80, 1.40)	<0.001
Glucose (mg/dL), median (IQR)	125.00 (105.00, 157.00)	123.00 (104.00, 153.00)	138.00 (112.00, 186.25)	<0.001
Magnesium (mg/dL), median (IQR)	2.00 (1.80, 2.20)	2.00 (1.80, 2.20)	1.90 (1.80, 2.10)	<0.001
Potassium (mg/dL), median (IQR)	4.20 (3.90, 4.50)	4.20 (3.90, 4.50)	4.15 (3.80, 4.60)	0.679
Sodium (mg/dL), median (IQR)	139.00 (137.00, 141.00)	139.00 (137.00, 141.00)	138.00 (136.00, 140.00)	<0.001
Urea nitrogen (mg/dL), median (IQR)	17.00 (14.00, 23.00)	17.00 (14.00, 22.00)	19.00 (14.00, 28.00)	<0.001
Hematocrit (%), median (IQR)	34.90 (30.10, 39.20)	34.60 (29.70, 38.90)	37.00 (32.40, 40.90)	<0.001
Hemoglobin (g/dL), median (IQR)	12.00 (10.19, 13.60)	11.90 (10.19, 13.50)	12.60 (10.90, 14.10)	<0.001
INR, median (IQR)	1.20 (1.10, 1.40)	1.20 (1.10, 1.40)	1.20 (1.10, 1.30)	<0.001
Platelet count (K/uL), median (IQR)	206.00 (162.00, 257.00)	201.00 (159.00, 250.00)	242.00 (195.00, 297.00)	<0.001
PT (s), median (IQR)	13.50 (12.70, 14.80)	13.50 (12.70, 14.80)	13.30 (12.40, 14.60)	<0.001
PTT (s), median (IQR)	30.50 (26.70, 39.00)	30.40 (26.80, 37.90)	30.65 (25.50, 57.50)	0.294
RDW (%), median (IQR)	13.60 (13.00, 14.20)	13.50 (13.00, 14.20)	13.90 (13.30, 15.03)	<0.001
Red blood cells (m/uL), median (IQR)	3.93 (3.36, 4.45)	3.89 (3.32, 4.42)	4.18 (3.59, 4.65)	<0.001
White blood cells (k/uL), median (IQR)	9.10 (7.00, 12.20)	8.90 (6.90, 12.00)	10.25 (8.30, 13.20)	<0.001
Ventilator (*n*) (%)				<0.001
No	738 (17.86)	297 (8.27)	441 (81.67)	
Yes	3395 (82.14)	3296 (91.73)	99 (18.33)	

CABG, coronary artery bypass grafting; PCI, percutaneous coronary intervention; IQR, interquartile-range; DSW divorced, separated, or widowed; BMI, body mass index; ECI, Elixhauser comorbidity index; SBP, systolic blood pressure; DBP, diastolic blood pressure; MBP, mean blood pressure; INR, international normalized ratio; PT, prothrombin time; PTT, partial thromboplastin time; RDW, red cell distribution width.

**Table 2:** Cox regression analyses of the relationship between BMI and all-cause mortality for patients after CABG.

Univariate				Model I			Model II		
BMI	HR (95%CI)		*P* value	HR (95%CI)		*P* value	HR (95%CI)		*P* value
Normal	Reference			Reference			Reference		
Underweight	2.8003	1.560–5.028	<0.001	3.38	1.872–6.103	<0.001	2.576	1.424–4.660	0.002
Overweight	0.7787	0.646–0.939	0.009	0.875	0.724–1.057	0.166	0.825	0.682–0.998	0.048
Obesity	0.8758	0.731–1.050	0.151	1.09	0.904–1.314	0.366	0.908	0.751–1.097	0.318
*P* for trend			0.178			0.275			0.294

Model I: adjust with age, sex, race, and marital status. Model II: adjust with age, marital status, length of stay, ECI, urine output, pH, potassium, PT, and RDW.

**Table 3:** Cox regression analyses of the relationship between BMI and all-cause mortality for patients after PCI.

Univariate				Model I			Model II		
BMI	HR (95% CI)		*P* value	HR (95% CI)		*P* value	HR (95% CI)		*P* value
Normal	Reference			Reference			Reference		
Underweight	0.911	0.415–2.000	0.817	1.027	0.446–2.365	0.950	0.390	0.166–0.916	0.031
Overweight	0.613	0.432–0.871	0.006	0.672	0.469–0.963	0.030	0.675	0.468–0.972	0.035
Obesity	0.591	0.404–0.865	0.007	0.742	0.498–1.108	0.144	0.680	0.458–1.009	0.056
*P* for trend			0.002			0.056			0.054

Model I: adjust with age, sex, race, and marital status. Model II: adjust with sex, ECI, urine output, heart rate, temperature, hematocrit, RDW, and ventilator.

## Data Availability

Data were fully available at https://physionet.org/content/mimiciii-demo/1.4/.

## Abbreviations

BMI: Body mass index
CABG: Coronary artery bypass grafting
PCI: Percutaneous coronary intervention
RCS: Restricted cubic splines
HIPAA: Health Insurance Portability and Accountability Act
SQL: Structured Query Language
WHO: World Health Organization
ECI: Elixhauser Comorbidity Index
RDW: Red blood cell distribution width
HR: Hazard ratio
CI: Confidence interval.

## Supplementary Materials

Supplementary Materials Table S1: selected variables by multivariate Cox regression analysis for CAGB patients. Table S2: selected variables by multivariate Cox regression analysis for PCI patients. Table S3: Cox regression analyses of the relationship between BMI quartiles and all-cause mortality for patients after CABG. Table S4: Cox regression analyses of the relationship between BMI quartiles and all-cause mortality for patients after PCI. Tables S5: subgroup analysis of the associations between BMI and all-cause mortality for patients after CABG. Tables S6: subgroup analysis of the associations between BMI and all-cause mortality of patients after PCI. Supplementary material related to this article can be found in Supplementary Tables S1-S6.
