# Supplementary material for: Obesity Paradox of All-Cause Mortality in 4,133 Patients Treated with Coronary Revascularization
Source: J Interv Cardiol. 2021 Nov 18;2021:3867735. doi: 10.1155/2021/3867735 (PMC8616700; doi:10.1155/2021/3867735)
Supplement: Supplementary Materials — Table S1: selected variables by multivariate Cox regression analysis for CAGB patients. Table S2: selected variables by multivariate Cox regression analysis for PCI patients. Table S3: Cox regression analyses of the relationship between BMI quartiles and all-cause mortality for patients after CABG. Table S4: Cox regression analyses of the relationship between BMI quartiles and all-cause mortality for patients after PCI. Tables S5: subgroup analysis of the associations between BMI and all-cause mortality for patients after CABG. Tables S6: subgroup analysis of the associations between BMI and all-cause mortality of patients after PCI. Supplementary material related to this article can be found in Supplementary Tables S1-S6. [file 3867735.f1.doc]

Table S1. Selected variables by multivariate Cox regression analysis for CAGB patients.

| Variable | Multivariate analysis | | |
| --- | --- | --- | --- |
|  | HR | 95%CI | P-value |
| length of stay | 1.055 | 1.043-1.067 | <0.001 |
| ECI | 1.039 | 1.030-1.047 | <0.001 |
| Urine output | 1.000 | 0.999-0.999 | <0.001 |
| PH | 0.210 | 0.056-0.790 | 0.021 |
| Potassium | 1.294 | 1.114-1.501 | 0.001 |
| PT | 1.025 | 1.001-1.049 | 0.042 |
| RDW | 1.187 | 1.130-1.246 | <0.001 |
| Age |  |  |  |
| youth | Reference |  |  |
| middle aged | 1.618 | 1.335-1.960 | <0.001 |
| the elderly | 2.325 | 1.851-2.921 | <0.001 |
| Marital status |  |  |  |
| Married | Reference |  |  |
| Unmarried | 1.419 | 1.110-1.814 | 0.005 |
| DSW | 1.227 | 1.025-1.468 | 0.026 |
| Other | 1.020 | 0.741-1.405 | 0.902 |
| BMI |  |  |  |
| Normal | Reference |  |  |
| Underweight | 2.576 | 1.424-4.660 | 0.002 |
| Overweight | 0.825 | 0.682-0.998 | 0.048 |
| Obesity | 0.908 | 0.751-1.097 | 0.318 |

Abbreviations: *HR* hazard ratio, *CI* confidence interval, *ECI* Elixhauser Comorbidity Index, *PT* prothrombin time, *RDW* red cell distribution width, *BMI* body mass index.

Table S2. Selected variables by multivariate Cox regression analysis for PCI patients.

| Variable | Multivariate analysis | | |
| --- | --- | --- | --- |
|  | HR | 95%CI | P-value |
| ECI | 1.040 | 1.028-1.053 | <0.001 |
| Urine output | 0.999 | 0.999-0.999 | <0.001 |
| HeartRate | 1.014 | 1.003-1.026 | 0.016 |
| Temperature | 0.745 | 0.586-0.948 | 0.017 |
| Hematocrit | 0.972 | 0.945-0.999 | 0.045 |
| RDW | 1.267 | 1.172-1.371 | <0.001 |
| Sex |  |  |  |
| Male | Reference |  |  |
| Female | 0.765 | 0.554-1.057 | 0.104 |
| BMI |  |  |  |
| Normal | Reference |  |  |
| Underweight | 0.390 | 0.166-0.916 | 0.031 |
| Overweight | 0.675 | 0.468-0.972 | 0.035 |
| Obesity | 0.680 | 0.458-1.009 | 0.056 |
| Ventilator |  |  |  |
| No | Reference |  |  |
| Yes | 1.710 | 1.206-2.426 | 0.003 |

Abbreviations: HR hazard ratio, CI confidence interval, ECI Elixhauser Comorbidity Index, RDW red cell distribution width, BMI body mass index.

Table S3.Cox regression analyses of the relationship between BMI quartiles and all-cause mortality for patients after CABG.

|  | Univariate | | |  | Model I | | |  | Model II | | |
| --- | --- | --- | --- | --- | --- | --- | --- | --- | --- | --- | --- |
| BMI | HR (95%CI) | | P |  | HR (95%CI) | | P |  | HR (95%CI) | | P |
| Q1 | Reference | | |  | Reference | | |  | Reference | | |
| Q2 | 0.722 | 0.586-0.888 | 0.002 |  | 0.759 | 0.616-0.936 | 0.010 |  | 0.740 | 0.599-0.913 | 0.005 |
| Q3 | 0.844 | 0.694-1.026 | 0.089 |  | 0.972 | 0.797-1.185 | 0.776 |  | 0.917 | 0.751-1.120 | 0.395 |
| Q4 | 0.821 | 0.672-1.001 | 0.051 |  | 1.025 | 0.834-1.260 | 0.812 |  | 0.828 | 0.672-1.021 | 0.078 |
| P for trend | |  | 0.112 |  |  |  | 0.546 |  |  |  | 0.212 |

Model I: Adjust with age, sex, race, and marital status.

Model II: Adjust with age, marital status, length of stay, ECI, urine output, PH, potassium, PT, and RDW.

Table S4. Cox regression analyses of the relationship between BMI quartiles and all-cause mortality for patients after PCI.

|  | Univariate | | |  | Model I | | |  | Model II | | |
| --- | --- | --- | --- | --- | --- | --- | --- | --- | --- | --- | --- |
| BMI | HR (95%CI) | | P |  | HR (95%CI) | | P |  | HR (95%CI) | | P |
| Q1 | Reference | | |  | Reference | | |  | Reference | | |
| Q2 | 0.692 | 0.473-1.013 | 0.058 |  | 0.745 | 0.504-1.102 | 0.141 |  | 0.914 | 0.618-1.353 | 0.654 |
| Q3 | 0.506 | 0.332-0.773 | 0.002 |  | 0.572 | 0.370-0.884 | 0.012 |  | 0.601 | 0.392-0.924 | 0.020 |
| Q4 | 0.576 | 0.383-0.866 | 0.008 |  | 0.701 | 0.458-1.075 | 0.103 |  | 0.987 | 0.644-1.513 | 0.953 |
| P for trend | |  | 0.002 |  |  |  | 0.037 |  |  |  | 0.313 |

Model I: Adjust with age, sex, race, and marital status.

Model II: Adjust with age, marital status, length of stay, ECI, urine output, PH, potassium, PT, and RDW.

Tables S5.Subgroup analysis of the associations between BMI and all-cause mortality for patients after CABG.

|  | No. of patients (%) | | BMI level | | | | P for interaction |
| --- | --- | --- | --- | --- | --- | --- | --- |
|  |  |  | Q1 | Q2 HR (95%CI) | Q3 HR (95%CI) | Q4 HR (95%CI) |  |
| Age, years | |  |  |  |  |  | 0.330 |
| ≤65 | | 1601 (44.6) | Reference | 0.703 (0.421, 1.173) | 1.246 (0.812, 1.912) | 0.759 (0.497, 1.158) |  |
| 66~79 | | 1567 (43.6) | Reference | 0.796 (0.584, 1.086) | 1.002 (0.755, 1.330) | 1.007 (0.745, 1.362) |  |
| ≥80 | | 425 (11.8) | Reference | 0.758 (0.529, 1.086) | 0.767 (0.494, 1.189) | 0.786 (0.490 1.260) |  |
| Sex | |  |  |  |  |  | 0.883 |
| Male | | 2751 (76.6) | Reference | 0.736 (0.573, 0.945) | 0.991 (0.779, 1.260) | 0.811 (0.623, 1.056) |  |
| Female | | 842 (23.4) | Reference | 0.757 (0.499, 1.149) | 0.878 (0.600, 1.285) | 0.885 (0.623, 1.258) |  |

Tables S6. Subgroup analysis of the associations between BMI and all-cause mortality of patients after PCI.

|  | No. of patients (%) | BMI level | | | | P for interaction |
| --- | --- | --- | --- | --- | --- | --- |
|  |  | Q1 | Q2 HR (95%CI) | Q3 HR (95%CI) | Q4 HR (95%CI) |  |
| Age, years |  |  |  |  |  | 0.343 |
| ≤65 | 246 (45.6) | Reference | 0.987 (0.412, 2.363) | 1.194 (0.494, 2.884) | 1.198 (0.518, 2.775) |  |
| 66~79 | 166 (30.7) | Reference | 1.406 (0.604, 3.276) | 0.552 (0.210, 1.450) | 1.239 (0.523, 2.934) |  |
| ≥80 | 128 (23.7) | Reference | 0.874 (0.453, 1.683) | 0.496 (0.244, 1.010) | 1.124 (0.483, 2.612) |  |
| Sex |  |  |  |  |  | 0.006 |
| Male | 362 (67.0) | Reference | 0.849 (0.507, 1.422) | 0.470 (0.270, 0.820) | 0.655 (0.351, 1.221) |  |
| Female | 178 (33.0) | Reference | 0.875 (0.442, 1.729) | 0.934 (0.467, 1.867) | 1.431 (0.778, 2.629) |  |
